# Supplementary material for: Defect engineering on V2O3 cathode for long-cycling aqueous zinc metal batteries
Source: Nat Commun. 2021 Nov 25;12:6878. doi: 10.1038/s41467-021-27203-w (PMC8617200; doi:10.1038/s41467-021-27203-w)
Supplement: Supplementary file 1 — Supplementary Information [file 41467_2021_27203_MOESM1_ESM.pdf]

Supplementary Information

Defect engineering on V_2O_3 cathode for long-cycling aqueous zinc metal batteries

Kefu Zhu[1,7], Shiqiang Wei[1,7], Hongwei Shou[1,7], Feiran Shen[2,7], Shuangming Chen[1,*], Pengjun Zhang[1], Changda Wang[1], Yuyang Cao[1], Xin Guo[1], Mi Luo[3], Hongjun Zhang[3], Bangjiao Ye[3], Xiaojun Wu[4], Lunhua He[2,5,6,*], Li Song[1,*]

[1] National Synchrotron Radiation Laboratory, CAS Center for Excellence in Nanoscience, University of Science and Technology of China, Hefei, 230029, China
[2] Spallation Neutron Source Science Center, Dongguan, 523803, China
[3] State Key Laboratory of Particle Detection and Electronics & Hefei National Laboratory for Physical Sciences at the Microscale, University of Science and Technology of China, Hefei, 230026, China
[4] School of Chemistry and Material Sciences, University of Science and Technology of China, Hefei, 230026, China
[5] Beijing National Laboratory for Condensed Matter Physics, Institute of Physics, Chinese Academy of Sciences, Beijing, 100190, China
[6] Songshan Lake Materials Laboratory, Dongguan, 523808, China
[7] These authors contribute equally.
***** Correspondence: Shuangming Chen (csmp@ustc.edu.cn),
Lunhua He (lhhe@iphy.ac.cn),
Li Song (song2012@ustc.edu.cn)

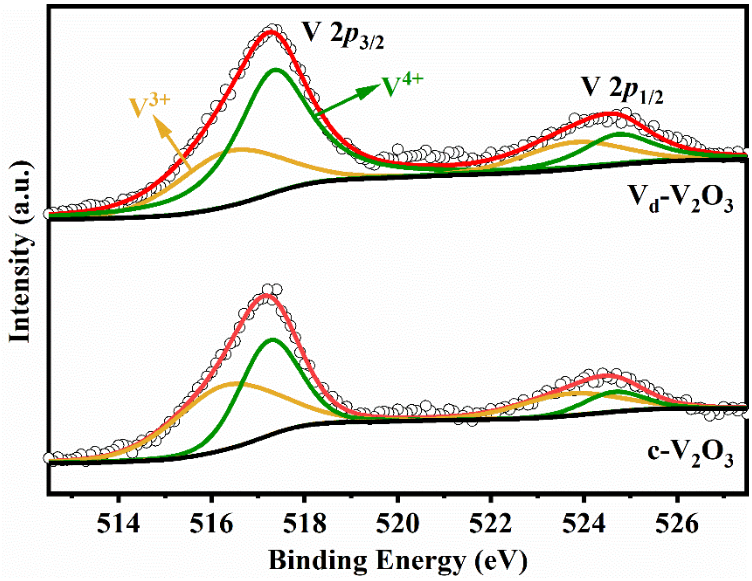

Supplementary Figure 1 | Comparison of the V 2*p* high-resolution XPS for V_d-V_2O_3 and c-V_2O_3.

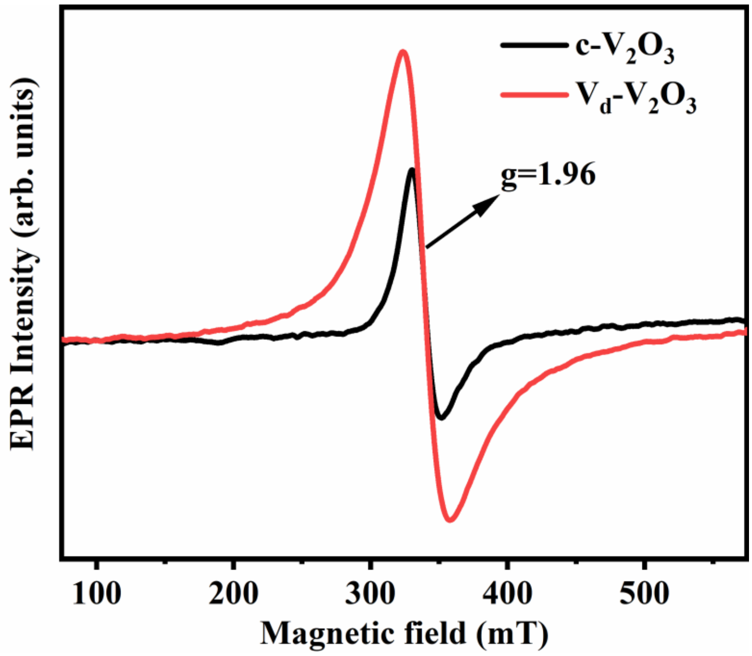

Supplementary Figure 2 | Comparison of EPR results for V_d-V_2O_3 and c-V_2O_3.

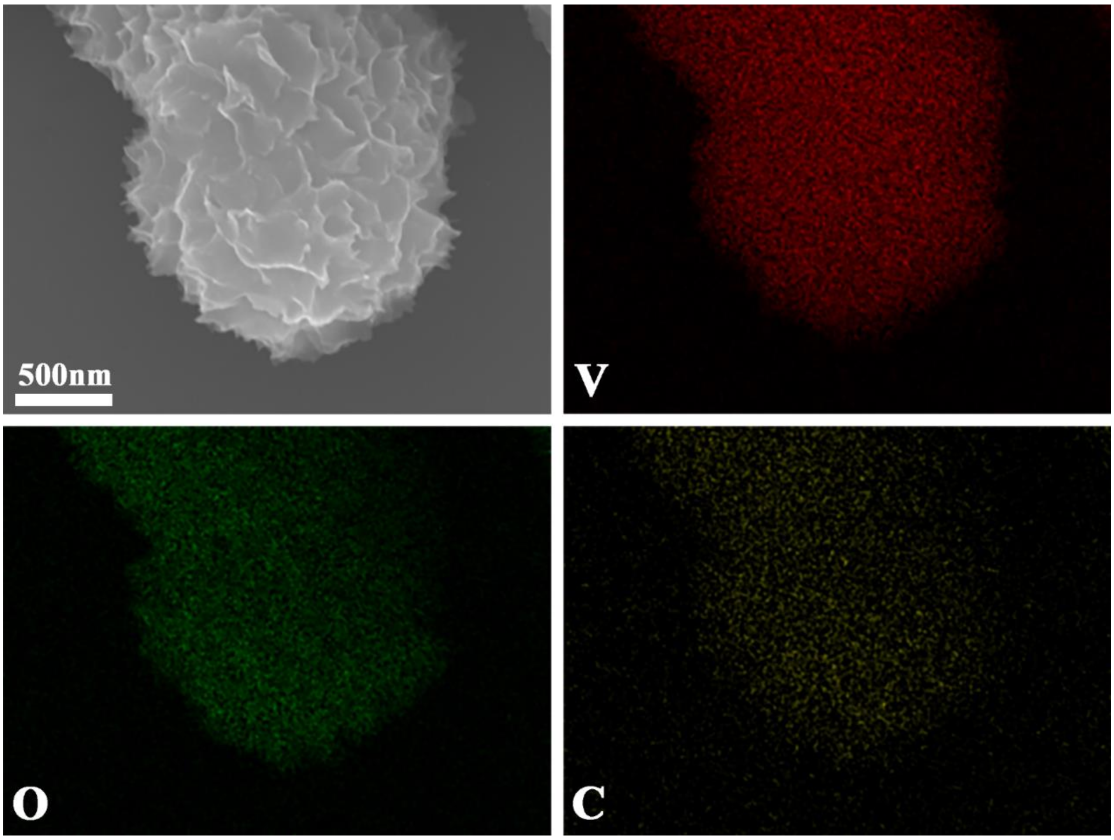

Supplementary Figure 3 | SEM element mapping images of V_d-V_2O_3.

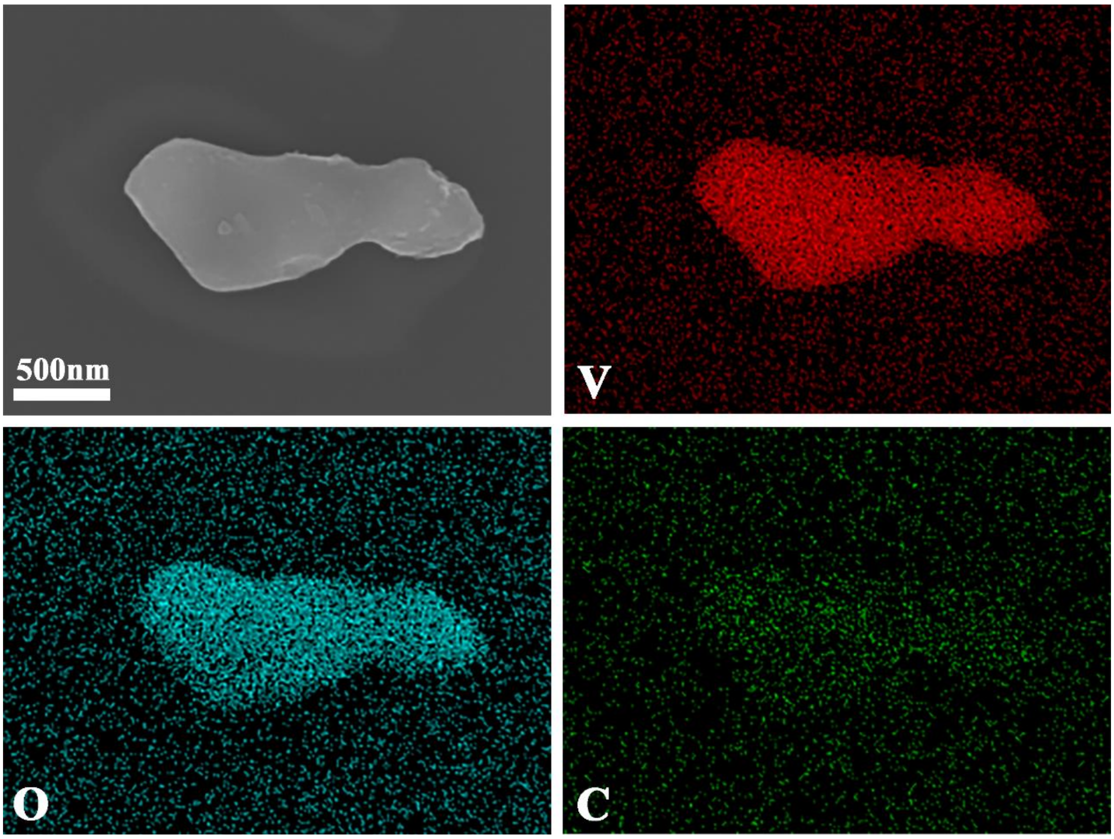

Supplementary Figure 4 | SEM element mapping images of c-V_2O_3.

The element mapping images of SEM of V_d-V_2O_3 and c-V_2O_3 both show that the V O elements are evenly distributed, but the C elements of V_d-V_2O_3 are significantly more than the c-V_2O_3.

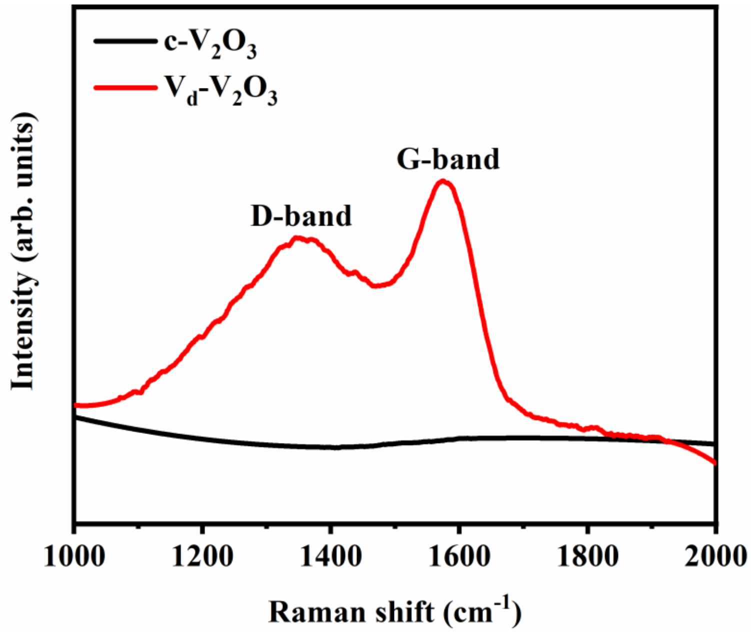

Supplementary Figure 5 | Raman spectra of the V_d-V_2O_3 and c-V_2O_3.

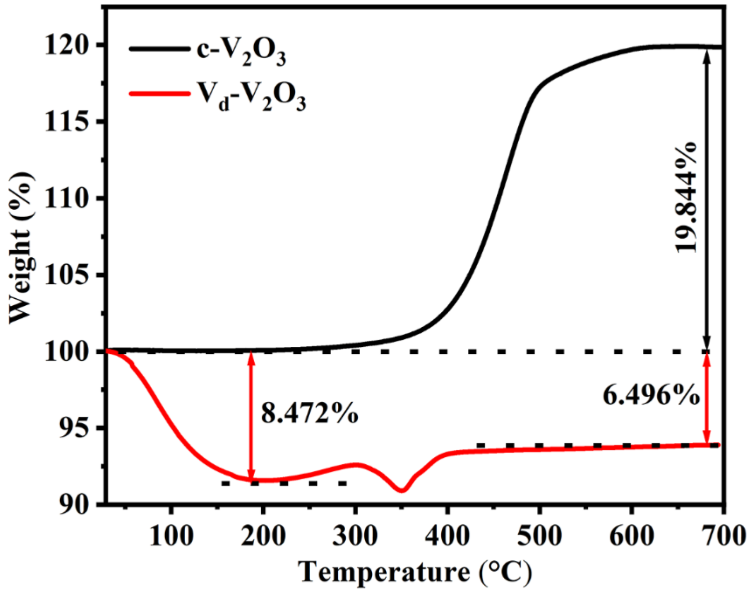

Supplementary Figure 6 | TGA curves of V_d-V_2O_3 and c-V_2O_3.

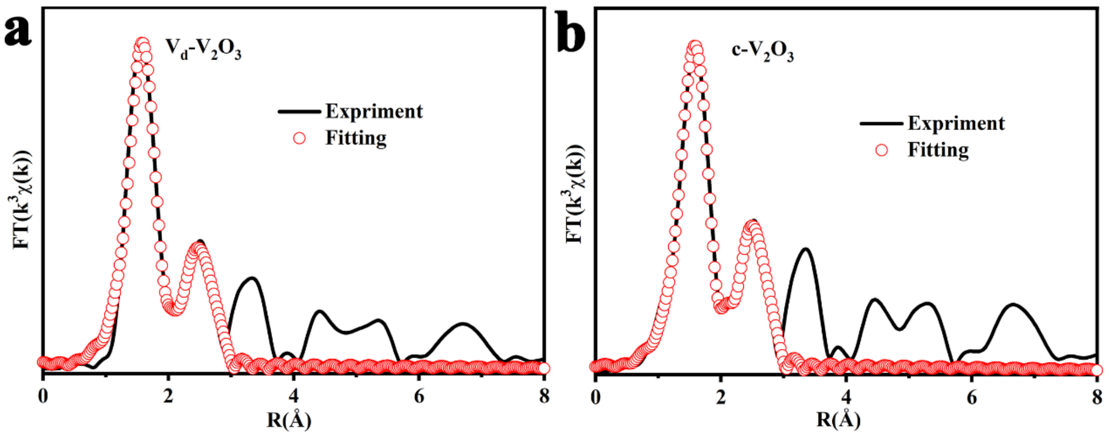

Supplementary Figure 7 | Experiment and fitted curves of k^3 weighted Fourier transform for V_d-V_2O_3 (a) and c-V_2O_3 (b).

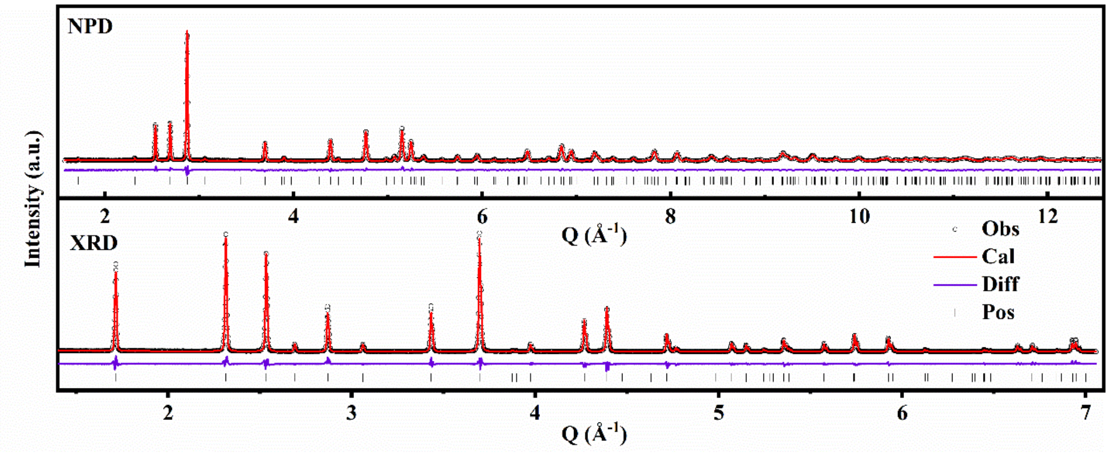

Supplementary Figure 8 | Neutron powder diffraction and X-ray diffraction refinement patterns for c-V_2O_3. Observed (black circle), calculated diffraction patterns (red line), their difference (purple line), and peak position (black bar) of the NPD pattern (upper part) and XRD pattern (lower part).

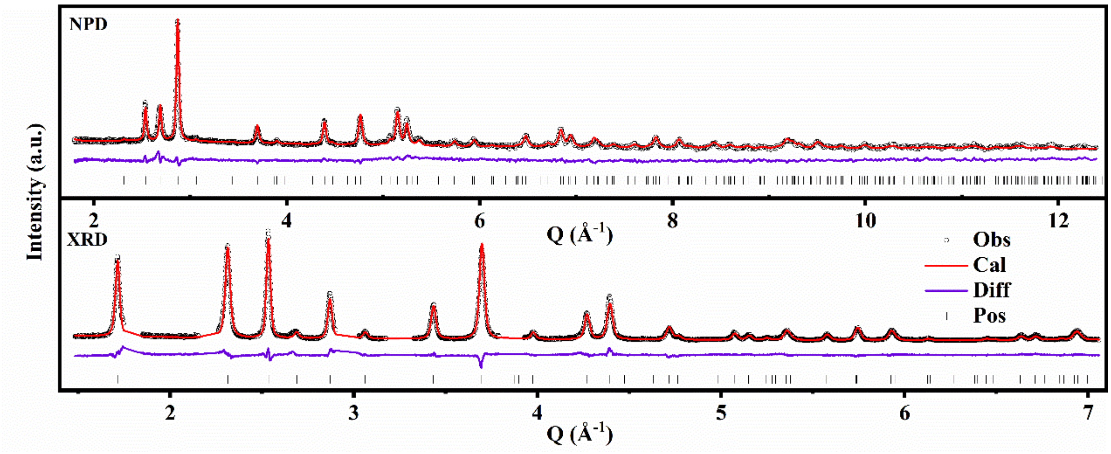

Supplementary Figure 9 | Neutron powder diffraction and X-ray diffraction refinement patterns for 0.5 h-V_d-V_2O_3. Observed (black circle), calculated diffraction patterns (red line), their difference (purple line), and peak position (black bar) of the NPD pattern (upper part) and XRD pattern (lower part).

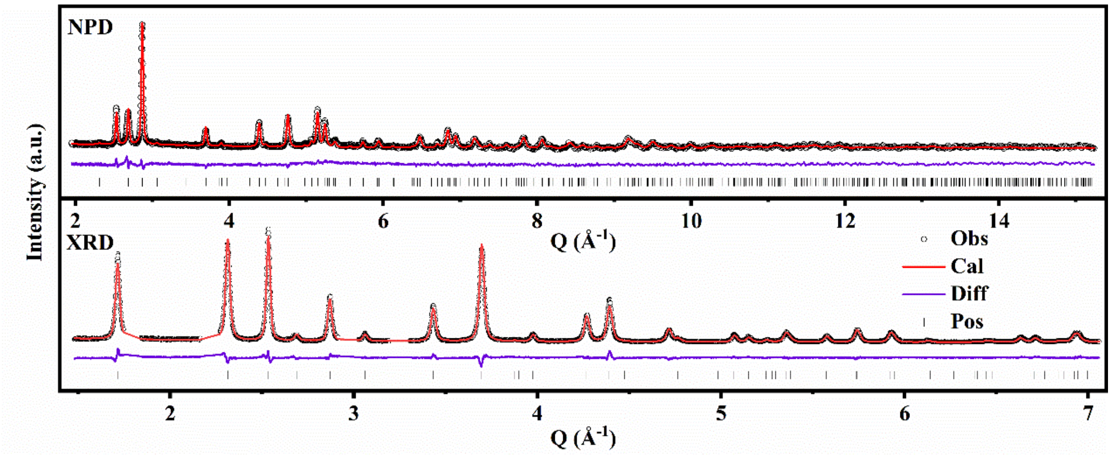

Supplementary Figure 10 | Neutron powder diffraction and X-ray diffraction refinement patterns for 6 h-V_d-V_2O_3. Observed (black circle), calculated diffraction patterns (red line), their difference (purple line), and peak position (black bar) of the NPD pattern (upper part) and XRD pattern (lower part).

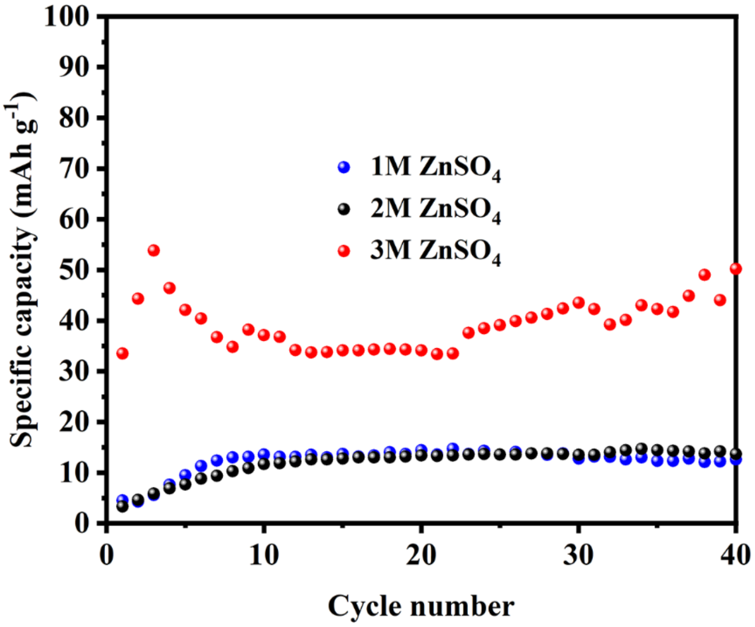

Supplementary Figure 11 | At 0.1 A g^{-1}, comparison of the discharge specific capacity for Zn||V_d-V_2O_3 cells in different concentrations of $ZnSO_4$ electrolyte.

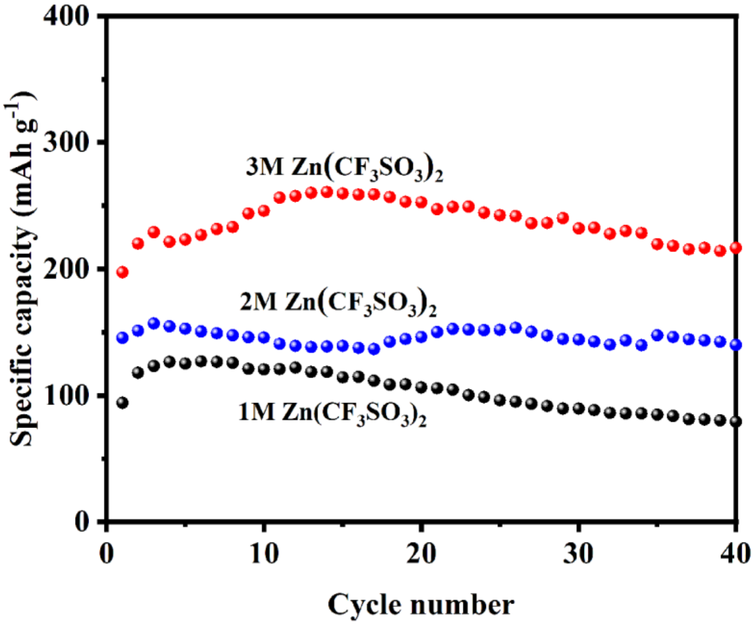

Supplementary Figure 12 | At 0.1 A g^{-1}, comparison of the discharge specific capacity for Zn||V_d-V_2O_3 cells in different concentrations of $Zn(CF_3SO_3)_2$ electrolyte.

Mild acid electrolytes such as $ZnSO_4$ solution[1,2] have inherently limited solubility and coulomb efficiency of Zn stripping/plating, while $Zn(CF_3SO_3)_2$ solution has high

ionic conductivity and electrochemical stability[3,4], and is widely used in aqueous zinc-ion batteries (ZIBs). Moreover, higher salt concentration can reduce the water activity and side reaction caused by water[5,6], thus improving the cycling stability of the electrode in aqueous solution. Accordingly, we investigated the battery performance of different concentrations of $ZnSO_4$ and $Zn(CF_3SO_3)_2$ and found that 3M $Zn(CF_3SO_3)_2$ as an aqueous electrolyte has the best cycle performance.

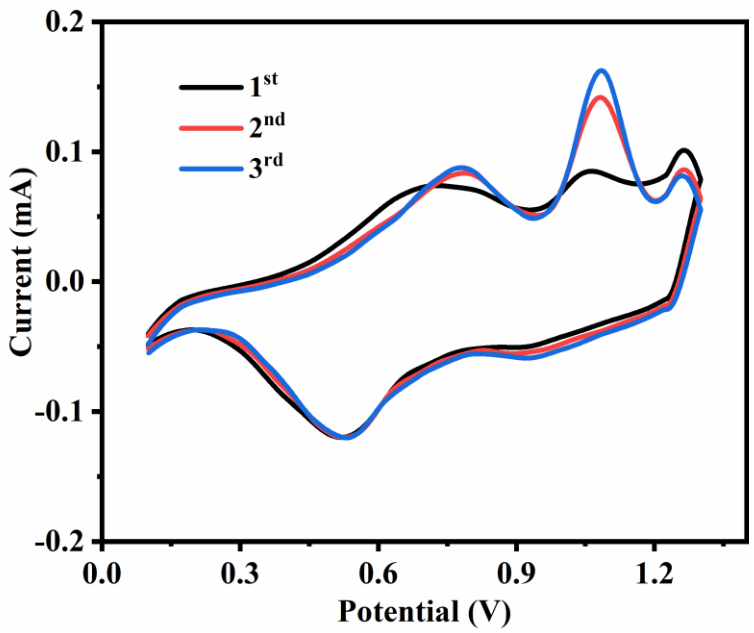

Supplementary Figure 13 | CV curves of the 1st, 2nd, and 3rd cycles for Zn||V_d-V_2O_3 cells at a scan rate of 0.1 mV s^{-1}.

The integral area of the first CV cycle is measurably smaller than the latter two cycles, suggesting upgraded electrochemical activity after the first cycle operation. This might be ascribed to an electrochemical activation process that produces a more eligible structure for Zn^{2+} intercalation. Subsequently, excellent reversibility is revealed by the overlapping CV curves of the last two cycles.

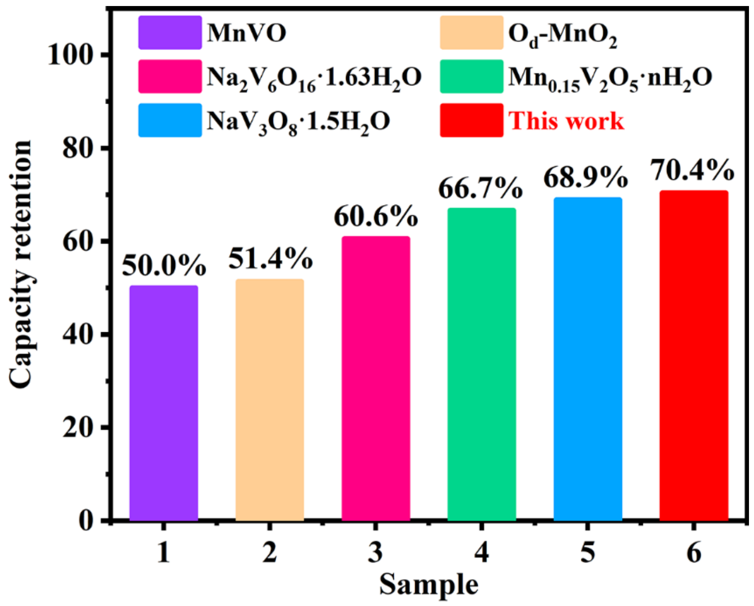

Supplementary Figure 14 | Comparison of the capacity retention between V_d-V_2O_3 and other vanadium-based cathodes for aqueous ZIBs.

We compared the capacity retention rate of different ZIBs electrodes when the specific current was increased by 10 times. The detailed ZIBs electrodes information is shown in **Supplementary Table 4**.

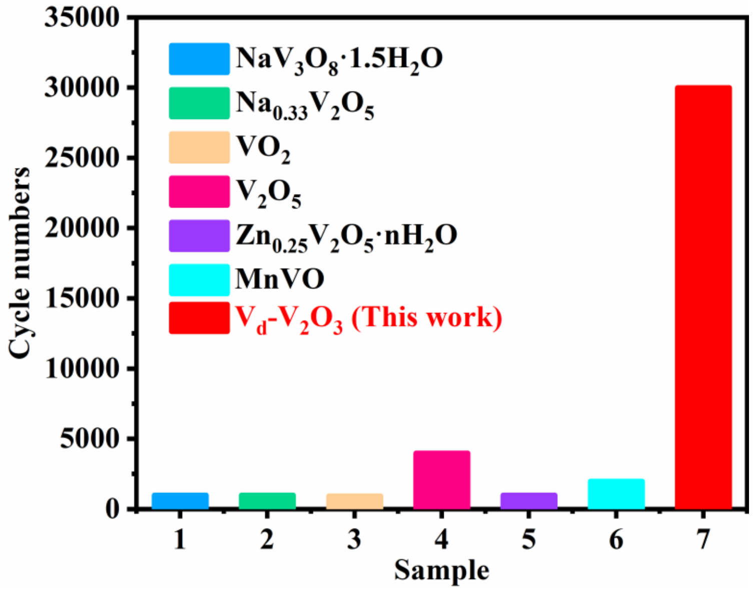

Supplementary Figure 15 | Comparison of the long-term cycling between V_d-V_2O_3 and other vanadium-based cathodes for aqueous ZIBs.

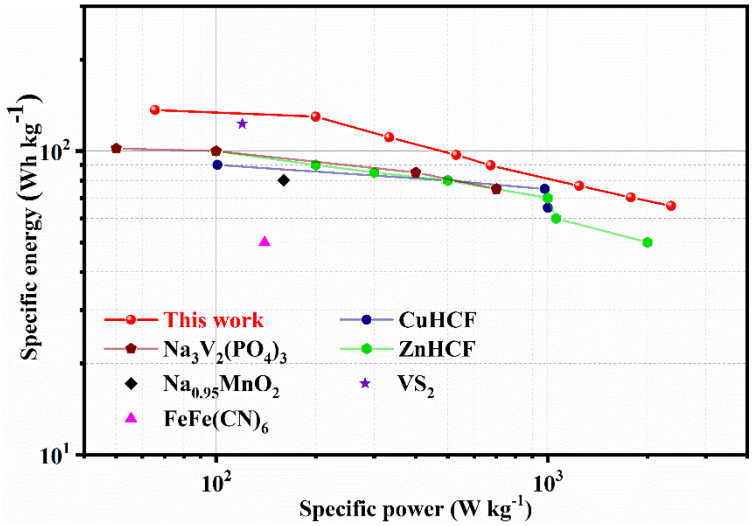

Supplementary Figure 16 | Ragone plot of the Zn||V_d-V_2O_3 cells and other aqueous Zn ion cells reported in the literature.

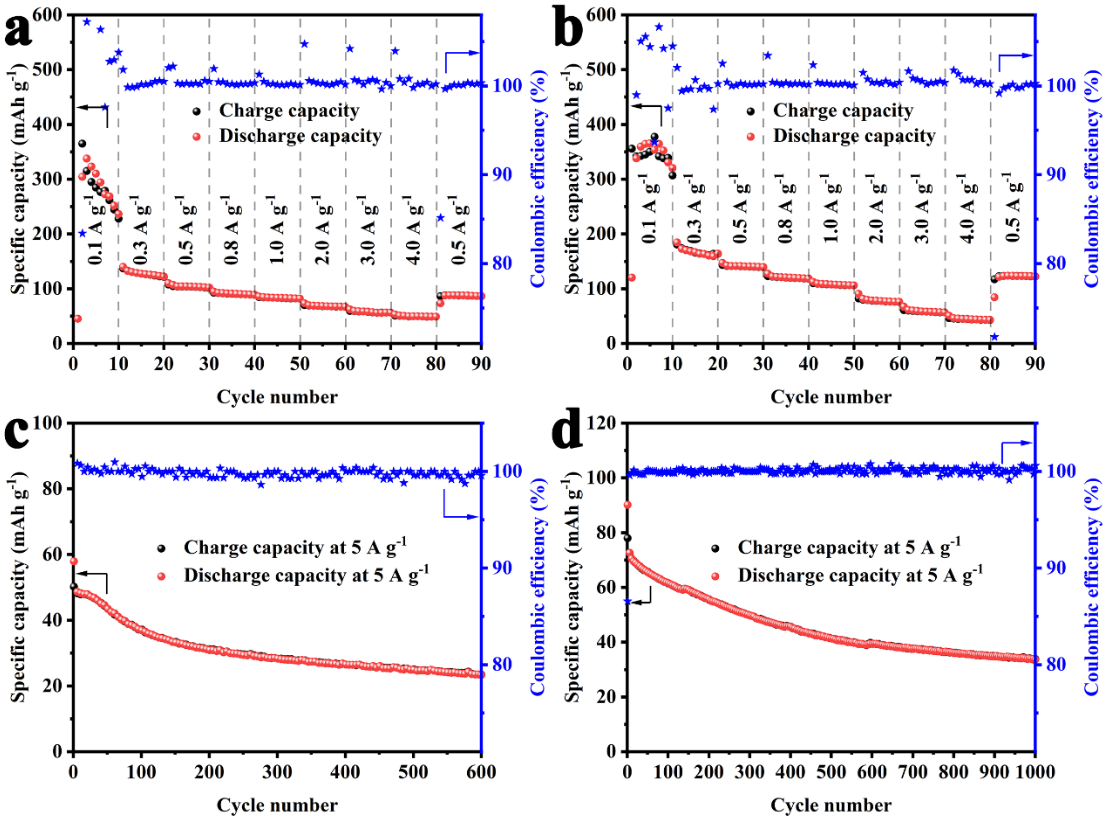

Supplementary Figure 17 | a, Rate performance at different specific current for 0.5 h-V_d-V_2O_3 electrode. **b**, Rate performance at different specific current of 6 h-V_d-V_2O_3 electrode. **c,** Cycling performance and coulombic efficiency of 0.5 h-V_d-V_2O_3 electrode at a specific current of 5 A g^{-1}. **d,** Cycling performance and coulombic efficiency of 6 h-V_d-V_2O_3 electrode at a specific current of 5 A g^{-1}.

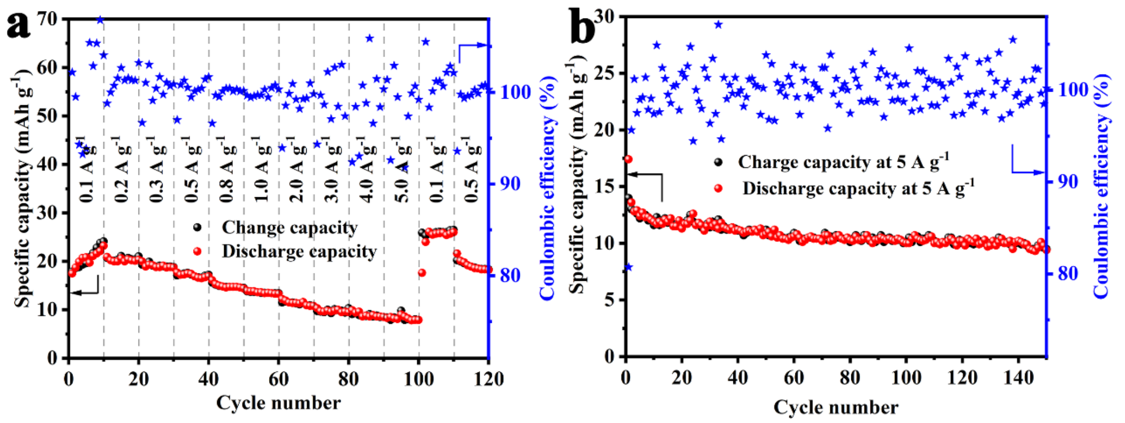

Supplementary Figure 18 | Electrochemical performance of c-V_2O_3 electrodes. (a) Rate performance under different specific current. (b) Cycling stability performance at 5 A g^{-1}.

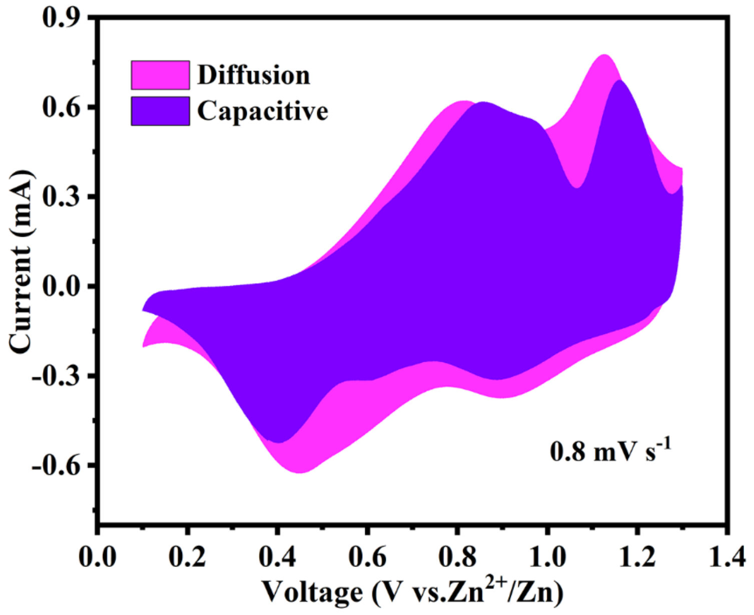

Supplementary Figure 19 | Contribution ratio of capacitance process (purple region) to total current at a scan rate of 0.8 mV s^{-1}.

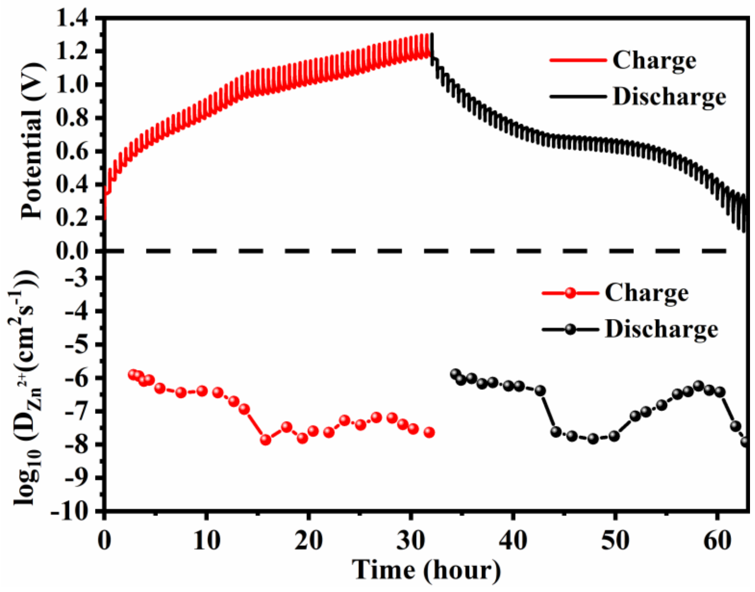

Supplementary Figure 20 | GITT curves and calculation of corresponding Zn^{2+} diffusion coefficient of Zn||V_d-V_2O_3 cell.

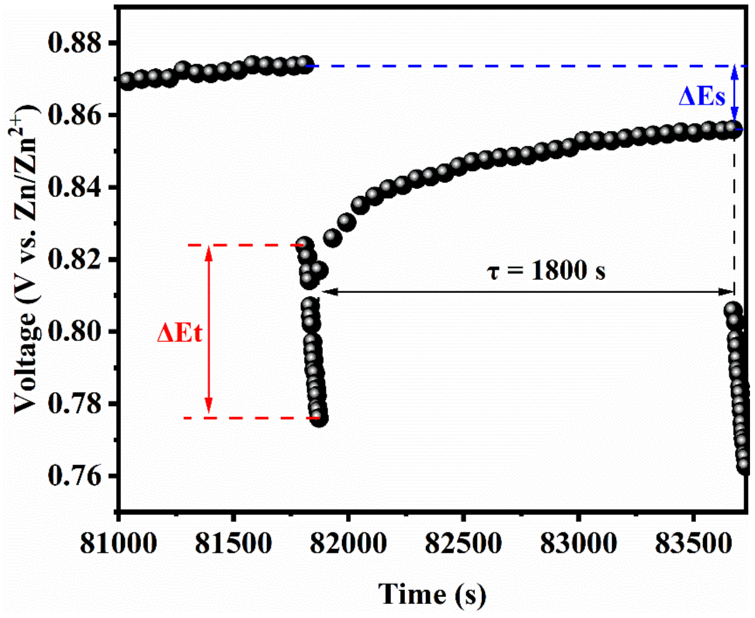

Supplementary Figure 21 | Schematic illustration of a selected single step of the GITT profile during charging.

The complete details of the calculation involved in estimating D_{Zn}^{2+} values are set out below.

The Galvanostatic Intermittent Titration Technique (GITT) was used to analyze the reaction and diffusion kinetics at 150 mA g^{-1} specific current, 1 min

charge/discharge time, and 30 min standing time. In the whole process of charge and discharge, the program is repeatedly applied to the battery. The diffusion coefficient of Zn^{2+} (D_{Zn}^{2+}, cm^{-2} s^{-1}) was calculated by GITT and based on the following formula:

$$D_{Zn} = \frac{4}{\pi\tau}\left(\frac{m_B V_m}{M_B A}\right)^2 \left(\frac{\Delta E_S}{\Delta E_\tau}\right)^2$$

Where τ represents the duration of the current pulse, m_B corresponds to the mass of the active material, M_B and V_B are related to the molecular mass (g mol^{-1}) and molar volume(cm^3 mol^{-1}), respectively. A is the total volume of the electrode in contact with the electrolyte, ΔE_S is the open-circuit voltage (V) difference measured at the end of two successive relaxation cycles, and ΔE_τ is the voltage change (V) during the constant current pulse **(Supplementary Figure 22)**.

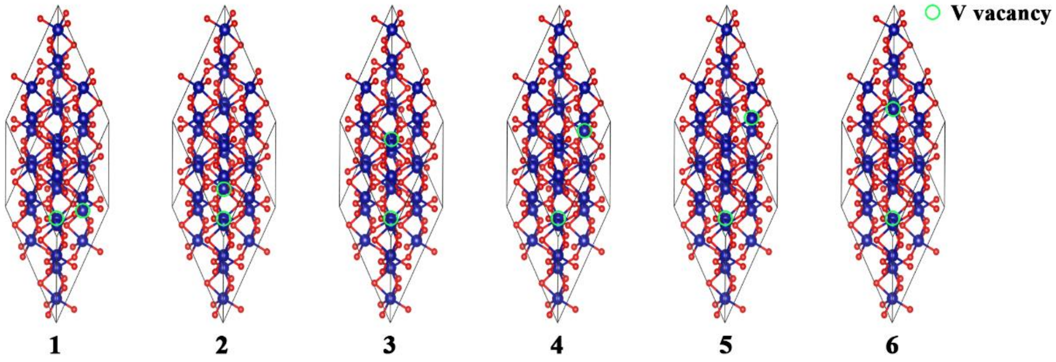

Supplementary Figure 22 | six random structure models of different vanadium vacancies in V_d-V_2O_3 at the concentration of 6.25%.

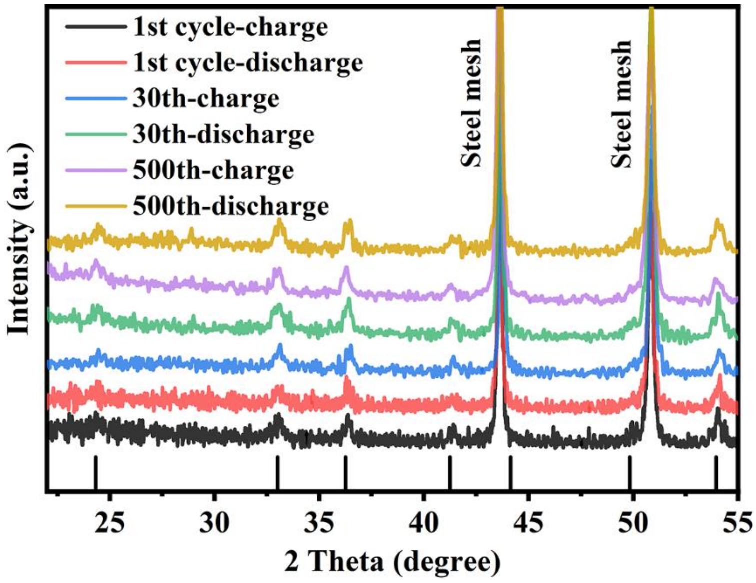

Supplementary Figure 23 | Ex situ XRD measurements of V_d-V_2O_3 electrodes under different cycles.

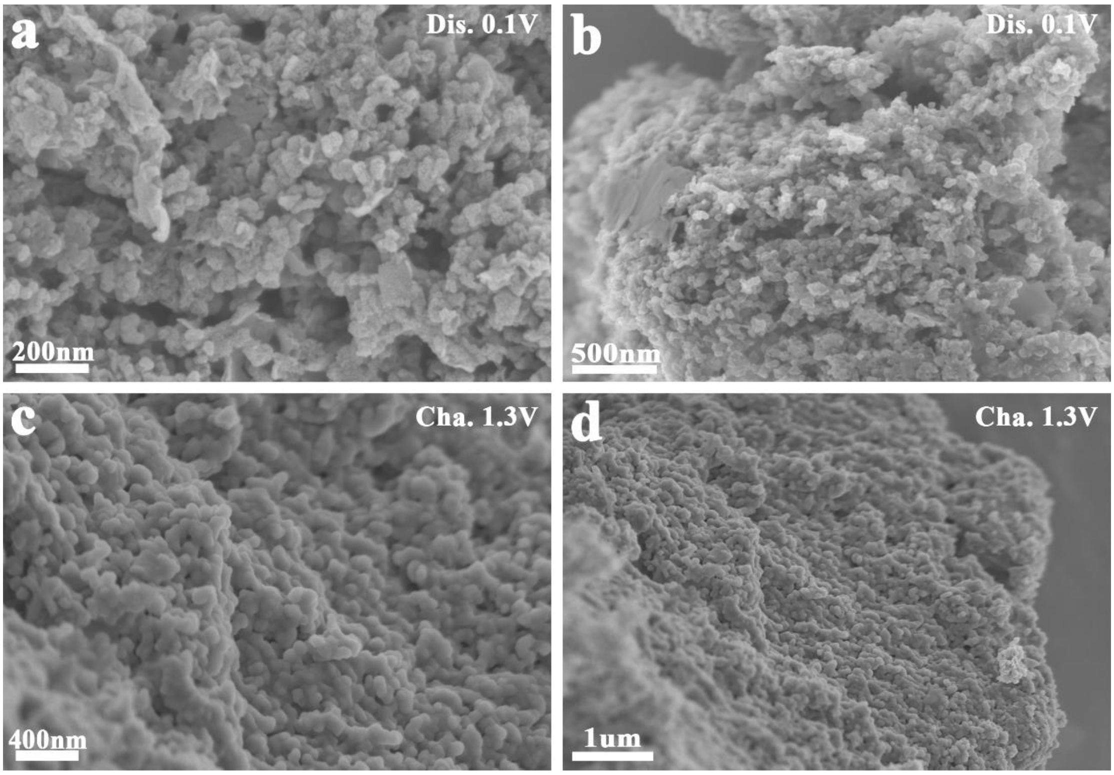

Supplementary Figure 24 | Ex situ SEM images of V_d-V_2O_3 electrodes at different voltages in 1st cycle.

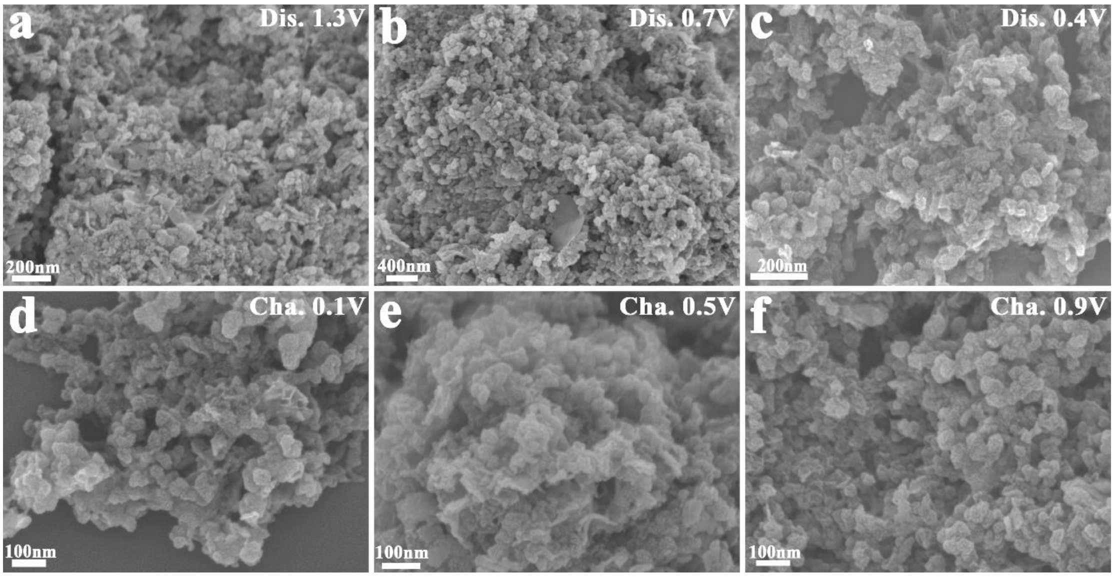

Supplementary Figure 25 | Ex situ SEM images of V_d-V_2O_3 electrodes at different voltages in 30th cycle.

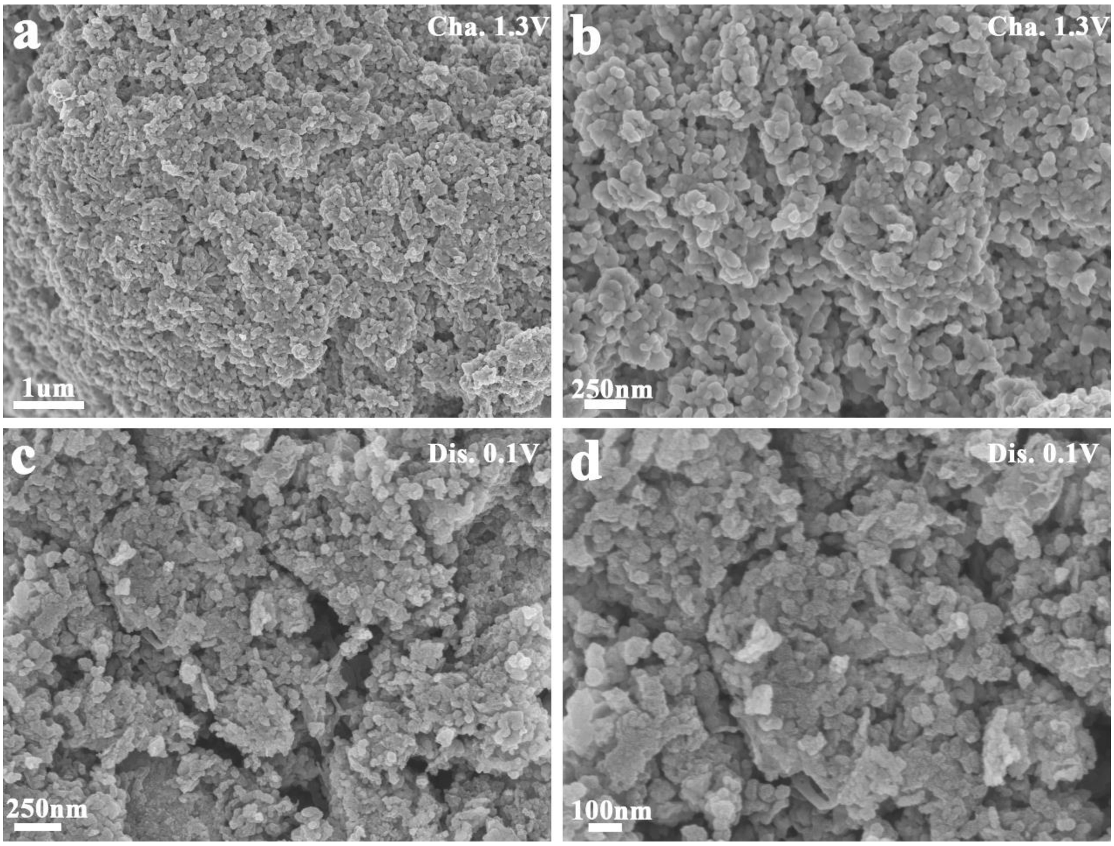

Supplementary Figure 26 | Ex situ SEM images of V_d-V_2O_3 electrodes at different voltages in 500th cycle.

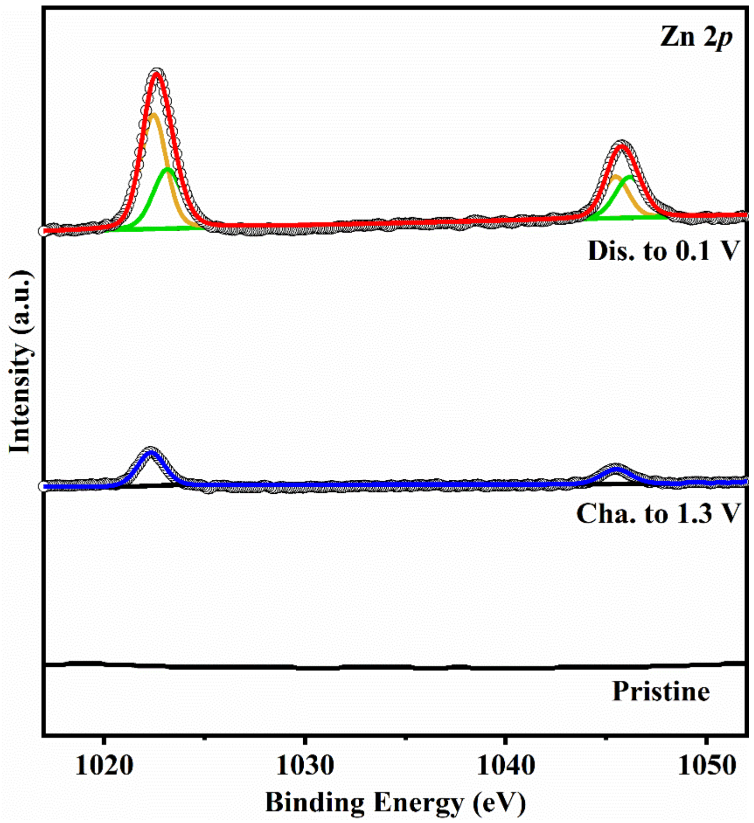

Supplementary Figure 27 | XPS high resolution spectrum of Zn 2*p* region.

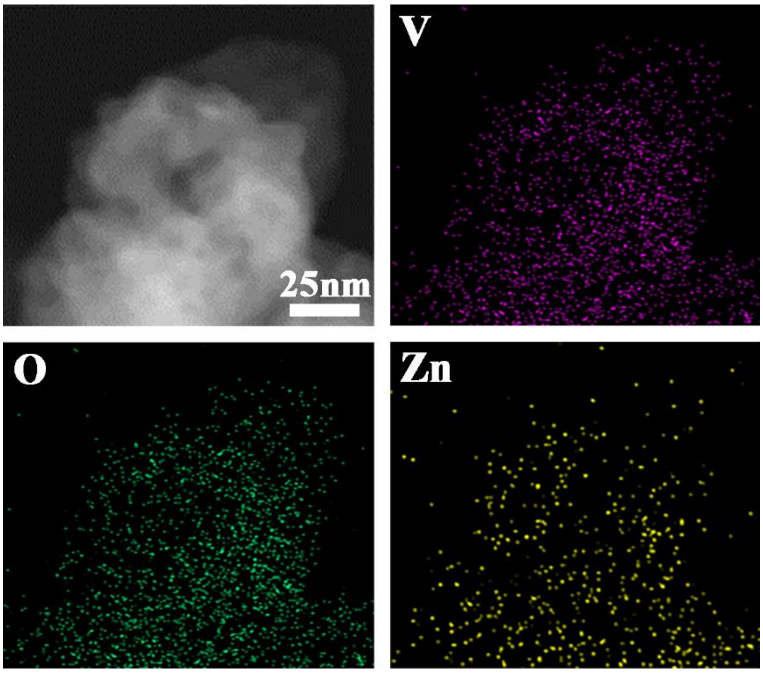

Supplementary Figure 28 | Ex situ TEM element mapping images of V_d-V_2O_3 after the 30 cycles of full charge.

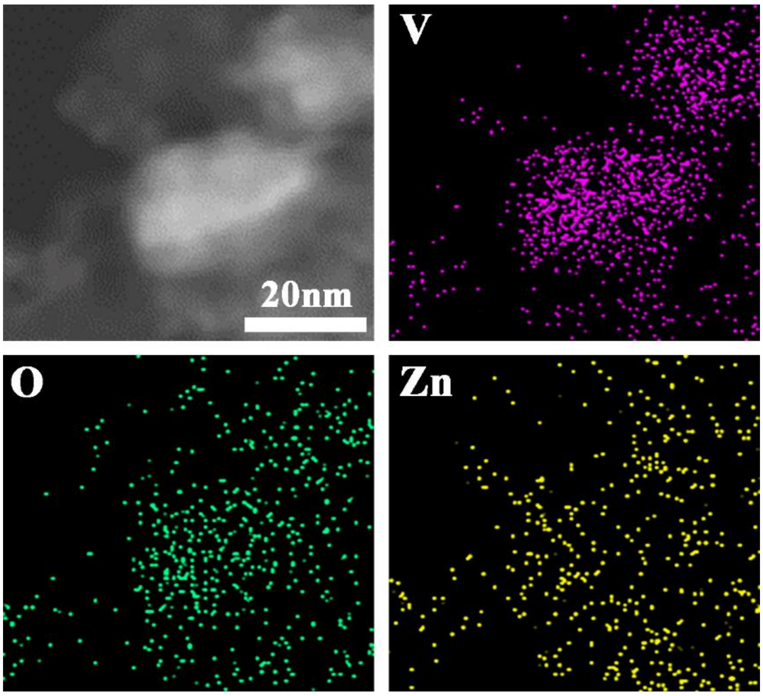

Supplementary Figure 29 | Ex situ TEM element mapping images of V_d-V_2O_3 after the 30 cycles of full discharge.

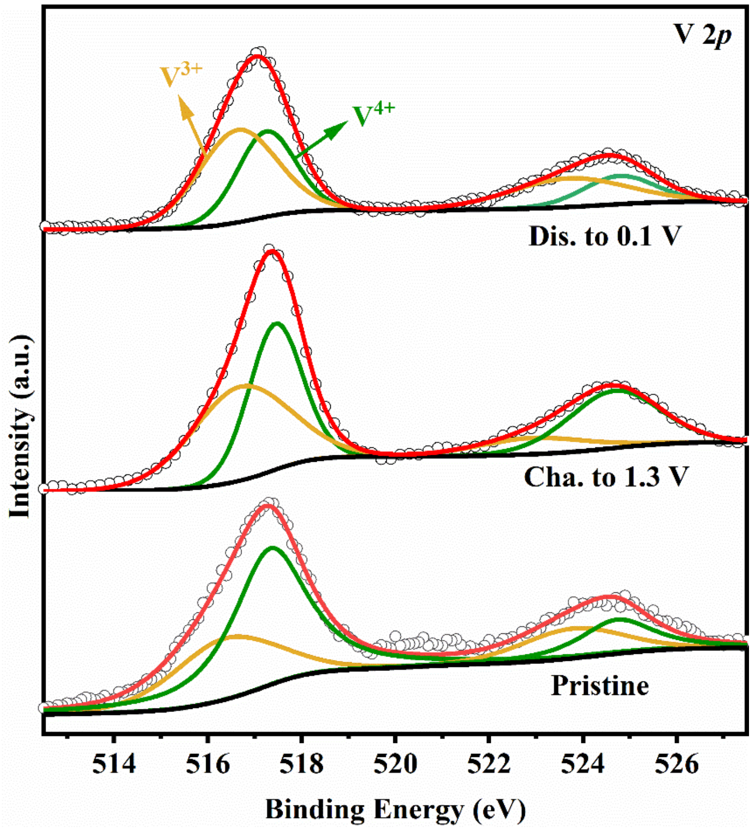

Supplementary Figure 30 | XPS high resolution spectrum of V 2*p* region.

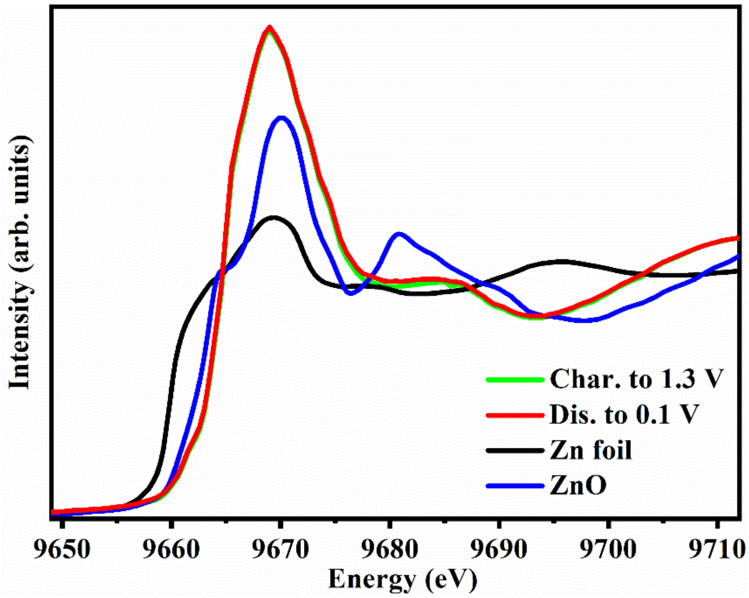

Supplementary Figure 31 | Normalized XANES spectrum of V_d-V_2O_3 under full charge/discharge, Zn foil and ZnO.

Supplementary Table 1 | XPS parameters of V_d-V_2O_3 and c-V_2O_3.

Sample	Position (eV)	Area	Area ratio
**V_d-V_2O_3**	516.280	15039.090	45.67%
	517.335	17887.550	54.32%
**c-V_2O_3**	516.296	15149.960	53.51%
	517.242	13162.830	46.49%

Supplementary Table 2 | Fitting parameters corresponding to V_d-V_2O_3 and c-V_2O_3.

Sample	Bond	N	R (Å)	σ^2 (10^{-3} Å^2)
**V_d-V_2O_3**	V-O	4.2	2.00	5.3
	V-V	0.6	2.75	3.4
	V-V	1.6	2.90	3.4
**c-V_2O_3**	V-O	6*	2.01	5.0
	V-V	1*	2.72	1.3
	V-V	3*	2.87	3.7

N, coordination number; σ^2, Debye-Waller factor; Error bounds (accuracies) were estimated as N, ±5%; Bond length, ±1%; σ^2, ±10%. * is fixed coordination number according to the standard crystal structure.

Supplementary Table 3 | Structural parameters from Rietveld refinement for V_d-V_2O_3.

Sample	Lattice parameters			Atomic occupancy		wRp	χ^2
				V	O		
**V_d-V_2O_3**	a=4.94734(9) Å<br>b=4.94734(9) Å<br>c=13.9990(5) Å	α=90°<br>β=90 °<br>γ=120 °	V=0.296 74 nm^3	94.3(1)%	102.7(3)% ≈ 100%	wRp(NDP) =2.95%<br>wRp(XRD) =3.6%	22.5
**c-V_2O_3**	a=4.95747(9) Å<br>b=4.95747(9) Å<br>c=14.01323(8) Å	α=90°<br>β=90 °<br>γ=120 °	V=0.298 26 nm^3	100.7(6)%	99.9(1)%	wRp(NDP) =3.76%<br>wRp(XRD) =13.77%	5.89
**0.5 h-V_d-V_2O_3**	a=4.95594(7) Å<br>b=4.95594(7) Å<br>c=14.0192(4) Å	α=90°<br>β=90 °<br>γ=120 °	V=0.298 20 nm^3	97.0(4)%	100.5(1)%	wRp(NDP) =3.58%<br>wRp(XRD) =12.55%	2.86
**6 h-V_d-V_2O_3**	a=4.95720(6) Å<br>b=4.95720(6) Å<br>c=14.0214(3) Å	α=90°<br>β=90 °<br>γ=120 °	V=0.298 40 nm^3	95.3(1)%	100.0(3)%	wRp(NDP) =3.71%<br>wRp(XRD) =10.72%	2.49

Supplementary Table 4 | Comparison of capacity retention for different vanadium-based aqueous ZIBs.

Sample	Specific Current (A g^{-1})	Electrolyte Concentration and Volume	Mass Loading	Testing Temperature	Cell Type	Capacity Retention Ratio(%)	Ref.
**MnVO**	0.2-2	3 M $Zn(CF_3SO_3)_2$, 130 uL	not mentioned	25 °C	coin cell	50.0	7
**O_d-MnO_2**	0.2-2	1 M $ZnSO_4$ with 0.2 M $MnSO_4$, not mentioned	1 mg	room temperature	coin cell	51.4	8
**$Na_2V_6O_{16}$·1.63H_2O**	0.1-1	3 M $Zn(CF_3SO_3)_2$, not mentioned	not mentioned	not mentioned	coin cell	60.6	9
**$Mn_{0.15}V_2O_5$·nH_2O**	0.5-5	1 M $Zn(ClO_4)_2$ in PC, not mentioned	1 mg cm^{-2}	not mentioned	coin cell	66.7	10
**C-KVO**	0.2-2	2 M $Zn(CF_3SO_3)_2$, not mentioned	1.5 mg cm^{-2}	room temperature	coin cell	68.9	11
**V_d-V_2O_3**	0.1-1	3 M $Zn(CF_3SO_3)_2$, 120 uL	1.13 mg cm^{-2}	25 °C	coin cell	70.4	This work

Supplementary Table 5 | Comparison of cycling stability for different vanadium-based aqueous ZIBs.

Sample	Electrolyte	Specific Current ($A\ g^{-1}$)	Reversible Capacity ($mA\ h\ g^{-1}$)	Cycle Number	Capacity Retention Ratio(%)	Ref.
**$NaV_3O_8\cdot$ 1.5 H_2O**	1 M $ZnSO_4$	4	140	1000	82	12
**$Na_{0.33}V_2O_5$**	3 M $Zn(CF_3SO_3)_2$	1	218.4	1000	93	13
**VO_2**	1 M $ZnSO_4$	3	165	945	75.5	14
**V_2O_5**	3 M $Zn(CF_3SO_3)_2$	5	372	4000	91.1	15
**$Zn_{0.25}V_2O_5\cdot$ n H_2O**	1 M $ZnSO_4$	2.4	200	1000	80	16
**MnVO**	3 M $Zn(CF_3SO_3)_2$	4	260	2000	92	17
**V_d-V_2O_3**	3 M $Zn(CF_3SO_3)_2$	5	117	10000	98	This work
**V_d-V_2O_3**	3 M $Zn(CF_3SO_3)_2$	5	102	20000	90	This work
**V_d-V_2O_3**	3 M $Zn(CF_3SO_3)_2$	5	97	30,000	81	This work

Supplementary Table 6 | Performance comparison of V_2O_3 under different vanadium vacancies concentrations.

Sample	Vanadium vacancies concentrations	Specific capacity at 0.3 A g^{-1}	Number of cycles at 5 A g^{-1}	Retention rate after cycle
V_d-V_2O_3	5.7%	187 mA h g^{-1}	30,000	81.0%
0.5 h-V_d-V_2O_3	4.7%	150 mA h g^{-1}	600	44.0%
6 h-V_d-V_2O_3	3.0%	184 mA h g^{-1}	1000	42.8%
c-V_2O_3	0.0%	18 mA h g^{-1}	150	76.9%

Supplementary Table 7 | The ICP-OES result of the V_d-V_2O_3 and c-V_2O_3 electrodes after the 30 cycles at different states.

Sample	Zn (ppm)	V (ppm)	Molar ratio (M_{Zn}/M_V)
V_d-V_2O_3 Char. to 1.3V	40.623	134.378	30.23
V_d-V_2O_3 Dis. to 0.1V	49.961	152.890	32.68
c-V_2O_3 Char. to 1.3V	19.364	195.521	9.90
c-V_2O_3 Dis. to 0.1V	22.326	195.503	11.42

Supplementary references

1. Alfaruqi, M. H. *et al.* Electrochemically induced structural transformation in a γ-MnO_2 cathode of a high capacity zinc-ion battery system. *Chem. Mater.* **27**, 3609-3620 (2015).
2. Lee, B. *et al.* Elucidating the intercalation mechanism of zinc ions into alpha-MnO_2 for

rechargeable zinc batteries. *Chem. Commun.* **51**, 9265-9268 (2015).
3. Zhang, N. *et al.* Cation-deficient spinel $ZnMn_2O_4$ cathode in $Zn(CF_3SO_3)_2$ electrolyte for rechargeable aqueous Zn-ion battery. *J. Am. Chem. Soc.* **138**, 12894-12901 (2016).
4. Ding, J. *et al.* Ultrafast Zn^{2+} intercalation and deintercalation in vanadium dioxide. *Adv. Mater.* **30**, 1800762 (2018).
5. Suo, L. *et al.* "Water-in-salt" electrolyte enables high-voltage aqueous lithium-ion chemistries. *Science* **350**, 938-943 (2015).
6. Wang, F. *et al.* Stabilizing high voltage $LiCoO_2$ cathode in aqueous electrolyte with interphase-forming additive. *Energy Environ. Sci.* **9**, 3666-3673 (2016).
7. Wei, S. et al. Manganese buffer induced high-performance disordered MnVO cathodes in zinc batteries. *Energy Environ. Sci.* **14**, 3954-3964 (2021).
8. Xiong, T. et al. Defect engineering of oxygen-deficient manganese oxide to achieve high-performing aqueous zinc ion battery. *Adv. Energy Mater.* **9**, 1803815 (2019).
9. Hu, P. et al. Highly durable $Na_2V_6O_{16}\cdot 1.63H_2O$ nanowire cathode for aqueous zinc-ion battery. *Nano Lett.* **18**, 1758-1763 (2018).
10. Geng, H. et al. Electronic structure regulation of layered vanadium oxide via interlayer doping strategy toward superior high-rate and low-temperature zinc-ion batteries. *Adv. Funct. Mater.* **30**, 1907684 (2019).
11. Yang, W. et al. 3D oxygen-defective potassium vanadate/carbon nanoribbon networks as high-performance cathodes for aqueous zinc-ion batteries. *Small Methods* **4**, 1900670 (2019).
12. Wan, F. et al. Aqueous rechargeable zinc/sodium vanadate batteries with enhanced performance from simultaneous insertion of dual carriers. *Nat. Commun.* **9**, 1656 (2018).
13. He, P. et al. Sodium ion stabilized vanadium oxide nanowire cathode for high-performance zinc-ion batteries. *Adv. Energy Mater.* **8**, 1702463 (2018).
14. Li, Z. et al. Mechanistic insight into the electrochemical performance of Zn/VO_2 batteries with an aqueous $ZnSO_4$ electrolyte. *Adv. Energy Mater.* **9**, 1900237 (2019).
15. Zhang, N. et al. Rechargeable aqueous $Zn-V_2O_5$ battery with high energy density and long cycle life. *ACS Energy Lett.* **3**, 1366-1372 (2018).
16. Kundu, D., Adams, B. D., Duffort, V., Vajargah, S. H. & Nazar, L. F. A high-capacity and long-life aqueous rechargeable zinc battery using a metal oxide intercalation cathode. *Nat. Energy* **1**, 16119 (2016).
17. Liu, C. et al. Expanded hydrated vanadate for high-performance aqueous zinc-ion batteries. *Energy Environ. Sci.* **12**, 2273-2285 (2019).
